# Data Fault Detection in Medical Sensor Networks

**DOI:** 10.3390/s150306066

**Published:** 2015-03-12

**Authors:** Yang Yang, Qian Liu, Zhipeng Gao, Xuesong Qiu, Luoming Meng

**Affiliations:** State Key Laboratory of Networking and Switching Technology, Beijing University of Posts and Telecommunications, No.10 Xitucheng Road, Haidian District, Beijing 100876, China; E-Mails: lq_1990@bupt.edu.cn (Q.L.); gaozhipeng@bupt.edu.cn (Z.G.); xsqiu@bupt.edu.cn (X.Q.); lmmeng@bupt.edu.cn (L.M.)

**Keywords:** fault detection, medical sensor, local outlier factor, fuzzy number

## Abstract

Medical body sensors can be implanted or attached to the human body to monitor the physiological parameters of patients all the time. Inaccurate data due to sensor faults or incorrect placement on the body will seriously influence clinicians’ diagnosis, therefore detecting sensor data faults has been widely researched in recent years. Most of the typical approaches to sensor fault detection in the medical area ignore the fact that the physiological indexes of patients aren’t changing synchronously at the same time, and fault values mixed with abnormal physiological data due to illness make it difficult to determine true faults. Based on these facts, we propose a Data Fault Detection mechanism in Medical sensor networks (DFD-M). Its mechanism includes: (1) use of a dynamic-local outlier factor (D-LOF) algorithm to identify outlying sensed data vectors; (2) use of a linear regression model based on trapezoidal fuzzy numbers to predict which readings in the outlying data vector are suspected to be faulty; (3) the proposal of a novel judgment criterion of fault state according to the prediction values. The simulation results demonstrate the efficiency and superiority of DFD-M.

## 1. Introduction

Wireless sensor networks (WSNs) have been widely used in medical applications. Body sensors are designed to be implanted in or adhere to the human body to monitor physiological parameters for a long term. For example, clinicians can monitor heart rate, blood glucose, or the body temperature of patients at any time [1,2]. Clinicians then diagnose the state of illness according to accurate sensed readings.

How to transmit and verify measurement data in a timely way has received more attention from researchers. The changing status of illness requires physiological readings to be reported frequently. What’s more, data inaccuracy due to limited resources (e.g., fluorescent lighting may cause data errors in pulse oximeters), or incorrect placement on the patient’s body will seriously influence the diagnosis of clinicians. e.g., piezoelectric sensors for snoring monitor the snore waveform of patients under sleeping, anaesthesized or sober conditions. The belt or medical adhesive tape may fall off and result in detection failure because patients always turn over.

Recently, some references have researched medical sensor detection problems [3,4,5]. They classify the states of patients as normal and abnormal, and then analyze readings’ abnormity according to intervals of physiological parameters to detect and isolate erroneous nodes, but these approaches have some drawbacks: (1) they detect data abnormities caused by sensor faults only when peoples’ states are stable, but it’s difficult to determine and judge when fault readings are mixed with abnormal physiological data; (2) in fact, physiological indicators of patients may be not be synchronously changing at the same time. For example, the heart rate can increase rapidly, while temperatures may slowly increase after a few minutes. Out-of-sync changes of medical attributes will cause a decrease in detection accuracy. Faulty measurements from sensors negatively influence the measured results and lead to diagnosis errors. Furthermore, they may threaten the life of a patient after alerting emergency personnel. Therefore, an important task is to detect abnormal and faulty measurements that deviate from real observations, and to distinguish sensor faults from real emergency situations in order to reduce false alarms.

In the paper, we mainly focus on detecting faulty medical sensors which generate faulty measurements mixed with abnormal physiological data, and accordingly increase the detection accuracy rate. We propose a Data Fault Detection in Medical body monitoring networks (DFD-M) method. Our innovations are:

(1) We firstly use a dynamic-LOF algorithm to identify outlying data vector. We find the new added objects which only influence parts of objects’ local outlier factors (LOF) in the original dataset. Finding these related objects whose LOF values have changed, the dynamic-LOF algorithm narrows down the scope of the detected objects’ nearest neighborhood to be more sensitive to outliers. What’s more, through finding three-levels of influenced objects, it can reduce the time complexity when the size of dataset is increasing dynamically.
(2) After locating an outlying sensed data vector, we use a fuzzy linear regression model based on trapezoidal fuzzy numbers to predict suspected readings in the data vector. In order to better fuzzify the prediction values, we construct an objective function of the sum of errors related to a fuzzy number expectation. These approaches help to embody a more precise relationship between prediction values and readings suspected to be faulty.
(3) Propose a novel judgment criterion for fault state according to the fuzzy prediction value. We summarize 15 relative position relationships between the fuzzy prediction results and the normal intervals of the corresponding physiological parameters, and accordingly conclude whether a sensor is at fault.

The rest of the paper is organized as follows: Section 2 describes some related works about fault detection in WSNs. Section 3 introduces the proposed DFD-M mechanism and involved algorithms, including dynamic-LOF, fuzzy linear regression process, and determination criterions. The simulation results in Section 4 demonstrate our algorithm’s efficiency and superiority. In Section 5, we conclude the paper.

## 2. Related Works

Typical faults in WSNs are link faults and data faults. For faulty link detection, transmission and reception will be influenced by destructive nodes. Link faults will cause network bottlenecks and partitioning. Most approaches use probes to fetch feedback information about faulty paths. Researchers analyze probe feedback according to network symptoms, and infer network topology and link states. A link scanner (LS) [6] collects hop counts of probe messages. It provides a blacklist containing all possible faulty paths. In probabilistic reasoning, each link generates two probe records. A link is deemed to fail to send probes when the collection record is mismatched with the expectation.

In istributed data fault detection, a sensor can determine its fault state through its neighbors’ monitoring values [7]. Sensors only know one-hop neighbors’ states instead of global information. Sensors should send their readings and determined tendency states periodically. When more than half of one-hop good neighbors are deemed it to be possibly good, then it maybe fault-free. The detection accuracy lies in the fault probability of sensors and network topology. Ding *et al.* [8] proposed a faulty identification method with lower computational overhead compared with [7]. If the difference between readings is large or large but negative, then the sensor is deemed as faulty.

Krishnamachari and co-workers proposed in [9] a distributed solution for the canonical task of binary detection of interesting environmental events. They explicitly take into account the possibility of measurement faults and develop a distributed Bayesian scheme for detecting and correcting faults. Each sensor node identifies its own status based on local comparisons of sensed data with some thresholds and dissemination of the test results [10]. Time redundancy is used for tolerating transient sensing and communication faults.

In the centralized mode, MANNA architecture [11] adopts a manager to control and manage a global vision of the wireless sensor network. Every node will check its energy level and send a message to the manager/agent whenever there is a state change. The manager obtains the coverage map and energy level of all sensors based upon the collected information. To detect node failures, the manager sends GET operations to retrieve the node state. Without hearing from the nodes, the manager will consult the energy map to check its residual energy. However, this approach requires an external manager to perform the centralized diagnosis and the communication between nodes and the manager is too expensive for WSNs.

In medical sensor networks, readings are typically accumulated and transmitted to a central device [4]. The device stores and processes data and judges illnesses. Focusing on diagnosis errors influenced by faulty measurements, [12] represents sensor fault and patient anomaly detection and classification. The approach classifies sensed readings into normal and abnormal, and then uses regression prediction to distinguish faulty readings from physiological abnormal readings. It can reduce the frequency of false alarm triggered by inconsistent monitoring. 

Reliable and light weight are also objectives in the detection of faults caused by sensors. The paper [13] proposes an online detection algorithm for isolating incorrect readings. To reduce false alarms, it firstly uses a discrete Haar wavelet decomposition and Hampel filter for detecting spatial deviations. Then a boxplot is adopted for temporal analysis. Miao *et al.* [14] presented an online lightweight failure detection scheme named Agnostic Diagnosis (AD), which is motivated by the fact that the system metrics of sensors usually exhibit certain correlation patterns. In [3], the authors present an online solution for fault detection and isolation of erroneous nodes in body sensor networks to provide sustained delivery of services despite encountered perturbations. It assumes that the sensors are either permanently faulty or fault free. Breunig *et al.* proposed an outlier detection method based on density and assign to each object a local outlier factor (LOF), which is the degree to which the object is outlying [15]. In [16], authors propose a fault detection and isolation algorithm in pervasive motion monitoring with two motion sensors, but in real life, sensors are not always perfectly synchronized. The poor constructions of sensors or monitoring program can generate missing data or unsynchronized data.

The dynamic changing illness of patients and out-of-sync variation of medical attributes influence the performance of data fault detection for medical sensors, so we further promote detection accuracy, reduce time complexity, and resolve the practical problem of out-of sync changing of sensed attributes.

## 3. Data Fault Detection of Body Sensors

We firstly assume physiological parameters have correlations between them. For example, heart rate (HR) is measured as the number of intervals in an electrocardiogram (ECG) signal. The respiration rate (RR) is proportional to the heart rate, but varies for different individuals. An additional one degree centigrade body temperature adds around 10–15 beats per minute to the heart rate [2]. In addition, episodes of low blood sugar (hypoglycemia) can trigger an arrhythmia, which often behaves like a racing heart [5]. In conclusion, respiration rate and body temperature are all positively correlated with heart rate.

Physiological parameters are correlated in time and space, and the correlation must be exploited to identify and isolate faulty measurements, in order to ensure reliable operation and accuracy diagnosis results. Usually, there is no spatial and temporal correlation among monitored attributes for faulty measurements. Based on the above theory, we introduce the data fault detection of body sensors, and focus on detecting faulty medical sensors so as to determine and judge the fault readings.

We firstly give some definitions. Physiological readings are described as a matrix *X* = (*X_ji_*), in which *j* represents measuring time, and *i* is sensor *i*. The sequence *X_i_* = (*X*_1*i*_, *X*_2*i*_, *X*_3*i*_, …, *X_ti_*) represents measured values of sensor *i* from time *T*_1_ to *T*_t_. The vector *X_j_* = (*X_j_*_1_, *X_j_*_2_, …, *X_jn_*) represents all of physiological parameters on time *T_j_*. 

The DFD-M mechanism is described as follows:To For reduce computational complexity and enhance detection average accuracy, a dynamic-LOF algorithm is applied in the increasing datasets. Based on this, it narrows down the scope of the detected objects’ nearest neighborhood to be more sensitive to outliers. Then, it finds out three levels of influenced objects and narrows the range of updating LOF values so as to improve the outlier detection efficiency (see Section 3.1).

Step 2: For an outlying vector, it needs to determine how many values are abnormal. Firstly, we define physiological normal and abnormal intervals of patients. For example, physiological normal HR is in the range (50, 130). Next, if all of readings are all in their normal or abnormal intervals and the corresponding vector is determined to be an outlier, no sensor is faulty, so this condition must be caused by a patient’s changing illness state. Otherwise, classify all of readings in the outlying vector into physiological normal and abnormal sets. The set which has more elements (supposed to be set *A*) will be regarded as reliable inputs of a fuzzy linear regression model. The outputs are the prediction values corresponding to elements in another set (supposed to be set *B*). As physiological indicators of patients may not synchronously changing at the same time, the prediction results only deduce that some sensors in set *B* are suspected to be faulty (see Section 3.2).

Step 3: For any reading *y* to be suspected, it must be a real number. While in the fuzzy regression process, the output is a fuzzy prediction value $\tilde{Y_{p}}=(y_{a},y_{b},y_{l},y_{r})$. Its lower bound is (*y_a_* − *y_l_*), and upper bound is (*y_b_* + *y_r_*). We need to analyze the difference between $\tilde{Y_{p}}$ and *y* according to the corresponding normal intervals. If *y* is closer to $\tilde{Y_{p}}$ according to the determination criterion (see Section 3.3), it means the sensor corresponding with the reading *y* is good, otherwise, it is faulty.

### 3.1. Dynamic-LOF Algorithm

An exact definition of an outlier depends on the data structure and detection methods. A data object is either an outlier or not. Breunig *et al.* proposed an outlier detection method based on density and assign to each object a local outlier factor (LOF), which is the degree to which the object is outlying [15]. It is local in that the degree depends on how isolated the object is with respect to the surrounding neighborhood, so the key idea of LOF is to compare the local density of an object’s neighborhood with that of its neighbors.

Our detection targets are dynamic time-series readings which are dynamic and constantly updated. Since the density-based LOF algorithm cannot detect contextual anomalies, the LOF values of all objects will be recalculated once the dataset changes. When the size of dataset increases, it will take a lot of time to frequently update the LOF values of all the objects in dataset, so the density-based LOF algorithm may not be suitable for the dynamic increment dataset. We find that newly added objects can only influence parts of objects’ LOF values in the original dataset. We only need to find out these related objects whose LOF values have changed, and recalculate their LOF values. Thus the efforts in recalculating LOF values of all the objects can be reduced, so in this paper, we propose a dynamic-LOF algorithm to identify outlying data vectors. We find out the three levels of influenced objects and recalculate these nodes’ new LOF values. Besides, a small modification is made to narrow down the scope of the detected objects’ nearest neighborhood, which can increase the detection accuracy, and then more outliers are detected.

The core idea of dynamic-LOF algorithm is, on the one hand, a small modification in k-distance, which makes our algorithm achieve higher detection average accuracy. On the other hand, finding out three levels of influenced objects and narrowing the range of updating LOF values to improve the outlier detection efficiency. The vector *X_j_* = (*X_j_*_1_, *X_j_*_2_, …, *X_jn_*) are all of sensed physiological readings at time *T_j_* is regarded as an object in multidimensional space. The size of dataset *D* continually increases over time. Firstly we obtain LOF values of all objects in dataset *D*, and then establish an initial knowledge base. All the objects in the initial knowledge base are considered normal. The dynamic-LOF algorithm only needs to update the LOF values of the newly added object and other objects which are influenced by the new one.

Assume *o*, *o'*, *p*, *q*, *s*, *x*, *y*, *z* to denote objects in a dataset *D*. Each object in dataset is assigned a local outlier factor. The larger the LOF is, the greater the chance is of an object being an outlier. We use the notation *d*(*s*, *o*) to denote the distance between objects *s* and *o*. *s* is an object in *D*. We take mean distance of object *s*, denoted as *mk-distance* to replace *k-distance* in the original LOF algorithm. It indicates the mean distance from *s* to its *k*-nearest objects.

*Definition 1.*  *k*-distance and *k*-distance neighborhood of an object *s*.

For any positive integer *k*, the *k-distance* of object *s*, denoted as *k-distance*(*s*) is defined as the distance *d*(*s*, *o*) between *s* and an object *o* ϵ *D* such that:

(1) For at least *k* objects *o'* ϵ *D*{*s*} it holds that *d*(*s*, *o'*) ≤ *d*(*s*, *o*), and
(2) For at most *k* − 1 objects *o'* ϵ *D*{*s*} it holds that *d*(*s*, *o'*) < *d*(*s*, *o*).

Then the *k*-distance neighborhood of *s* is *N_k_*(*s*) = {*q* ϵ *D*\{*s*}|*d*(*s*, *q*) ≤ *k-distance*(s)}. These objects *q* are called the *k*-nearest neighbors of *s*.

*Definition 2.*  mk-distance of an object s.

For any positive integer *k*, the *mk-distance* of object *s* is:

(1) $$mk-distance(s)=\frac{\sum_{o\in N_{k}(s)}d(s,o)}{\left|N_{k}(s)\right|}$$

*Definition 3.*  *mk-distance* neighborhood of an object s.

Given *mk*-distance of *s*, the *mk*-distance neighborhood of *s* contains every object whose distance from *s* is not greater than *mk-distance*, *i.e.*, *N_mk_*(*s*) = {*q* ϵ *D*\{*s*}|*d*(*s*, *q*) ≤ *mk-distance*(s)}. Each object *q* in *N_mk_*(*s*) is also in *N_k_*(*s*).

*Definition 4.*  Reachability distance of an object *s* with respect to object *o* is:

(2) $$r-dist_{mk}(s,o)=max\{mk-distance(o),d(s,o)\}$$

*Definition 5.*  Local reachability density of an object s is:

(3) lrdmk(s)=1(∑o∈Nmk(s)r−distmk(s,o)|Nmk(s)|)

*Definition 6.*  Local outlier factor of an object s is:

(4) LOFmk(s)=∑o∈Nmk(s)lrdmk(o)lrdmk(s)|Nmk(s)|

The outlier factor of object s captures the degree to which we call *s* an outlier. It is the average of the ratio of the local reachability density of *s* and those of its *mk*-distance neighbors. It is easy to see that the lower local reachability density is, and the higher the local reachability densities of its *mk*-distance neighbors are, the higher is the LOF value of *s*.

By using the above formulas to calculate the LOF*_mk_* values of all objects in the dataset, the scope of nearest neighborhood of each object can be narrowed down, so our improved algorithm is more sensitive to outliers, and can achieve higher detection average accuracy.

According to the steps above, it’s clear that the LOF value of each object depends on the local reachability density and its *k*-nearest neighbors. When there are any newly added, deleted, or updated objects, the LOF values of partially related objects would be influenced. In the environment of dynamic increment dataset, updating the LOF values of all the objects in dataset frequently will cost a great deal of temporal and spatial resources, but we note that only part of the related objects will be influenced by the changes of dataset, so we only need to find out these related objects whose LOF values have changed, and recalculate LOF values of these objects. Assume that an added object is *p*. According to Definition 1 to Definition 6, we also have the following definitions to find the three levels of influenced objects:

*Definition 7.*  The first-level influenced objects. Given a new added object *p*, the first-level influenced objects of *p* contains every object *x* whose distance from *p* is not greater than *k*-*distance*(*x*), and the first-level influenced objects can be defined as:

(5) $$F_{1}(p)=\{x|(x\in D\backslash \{p\})\wedge d(x,p)\leq k-distance(x)\}$$

The new object *p* makes *N_k_*(*x*) change, and then leads to the subsequent change of *N_mk_*(*x*), *lrd_mk_*(*x*) and *LOF_mk_*(*x*).

*Definition 8.*  The second-level influenced objects. The second-level influenced objects of *p* contains every object *y* whose distance from *x* (object in *F*_1_(*p*)) is not greater than *mk*-*distance*(*y*), and the second-level influenced objects can be defined as:

(6) $$F_{2}(p)=\{y|(y\in D\backslash F_{1}(p))\wedge (x\in F_{1}(p))\wedge d(y,x)\leq mk-distance(y)\}$$

These second-level influenced objects remain *N_mk_*(*y*) unchanged, but should recalculate *lrd_mk_*(*y*) and *LOF_mk_*(*y*) due to the change of *mk*-*distance*(*x*).

*Definition 9.*  The third-level influenced objects. The third-level influenced objects of *p* contains every object *z* whose distance from *y* (object in *F*_2_(*p*)) is not greater than *mk*-*distance*(*z*), and the third-level influenced objects is:

(7) $$F_{3}(p)=\{z|(z\in D\backslash \{F_{1}(p)\cup^{\text{​}}F_{2}(p)\})\wedge (y\in F_{2}(p))\wedge d(z,y)\leq mk-distance(z)\}$$

The third-level influenced objects only *LOF_mk_*(*z*) changed. Based on the abovementioned analysis, we find that because of the addition of object *p*, there are only three levels influenced objects need to recalculate their *LOF_mk_* values. Other objects’ *LOF_mk_* values remain unchanged.

*Definition 10.*  The set of influenced objects whose *LOF_mk_* values to be recalculated is *F*.

(8) $$F=F_{1}(p)\cup^{\text{​}}F_{2}(p)\cup^{\text{​}}F_{3}(p)$$

In the Dynamic LOF, we firstly obtain the *k*-distance neighborhood *N_k_*, mk-distance neighborhood *N_mk_*, local reachability density *lrd_mk_* and local outlier factor LOF*_mk_* of all the objects in dataset *D*. We put the new objects into the knowledge base, and find in turn the three level influenced objects based on the new knowledge base. Finally, we update *N_k_*, *N_mk_*, *lrd_mk_* and LOF*_mk_* of these objects, while the LOF*_mk_* values of other objects remain unchanged. Finally, the LOF*_mk_* value of each incoming new object will be calculated according to the unceasing updating of the knowledge base. If the LOF*_mk_* value of a new object is smaller than a given threshold (an empirical value, equal to 2.0), it is normal. Otherwise it is outlying. Algorithm 1 describes the process of finding the first-level influenced objects after adding a new object *p* into dataset *D*.

**Algorithm 1.** Find First Level Objects (*D*, *F*_1_(*p*), *p*).0: Initialize $F_{1}(p)$ 1: **for**
d(x,p)≤k−distance(x)
**do** // The new object *p* is in the *k*-distance neighborhood of object *x* 2: input *x* into $F_{1}(p)$; // Construct the set of the first-level influenced objects 3: **end for** 4: input *p* into *D* 5: input *p* into $F_{1}(p)$ // *p* is also contained in $F_{1}(p)$

*F*_1_(*p*) indicates the set of the first-level influenced objects (including *p*). If *p* is in the *k*-distance neighborhood of object *x*, then the object *x* is a first-level influenced object, and should be put into *F*_1_(*p*). In the end, all of objects in *F*_1_(*p*) should be recalculated the values of *N_k_*, *N_m__k_*, *lrd_mk_* and LOF*_mk_*, so does object *p*, so we also put object *p* into *F*_1_(*p*). Algorithms 2 and 3 describe the process of constructing the sets of *F*_2_(*p*) and *F*_3_(*p*).

**Algorithm 2.** Find Second Level Objects (*D*, *F*_2_(*p*), *F*_1_(*p*)) 0: Initialize $F_{2}(p)$ 1: **for** all objects *x* in $F_{1}(p)$\{*p*} **do** 2: **for** all objects *y* in D\$F_{1}\left(p\right)$ && *x* is in $N_{mk}(y)$
**do** 3: input *y* into $F_{2}(p)$ ; // Construct the set of the second-level influenced objects 4: **end for** 5: **end for**

**Algorithm 3.** Find Third Level Objects (*D*, $F_{3}(p)$, $F_{2}(p)$).0: Initialize $F_{3}(p)$ 1: **for** all objects *y* in $F_{2}(p)$
**do** 2: **for** all objects *z* in D\{$F_{1}(p)\cup^{\text{​}}F_{2}(p)$} && *y* is in $N_{mk}\left(z\right)$
**do** 3: input *z* into $F_{3}(p)$; // Construct the set of the third-level influenced objects 4: **end for** 5: **end for**

Algorithm 4 describes the update process of $N_{k}(x)$ and $N_{mk}(x)$, where *x* is a first-level influenced object.

**Algorithm 4**. Update K-Distance ($F_{1}(p)$).0: **for** all objects *x* in $F_{1}(p)$\{*p*} **do** 1:  ** if**
d(x,p)=k−distance(x)
**then** 2:  input *p* into $N_{k}(x)$ ; 3:  **else if** (d(x,p)<k−distance(x) && there are less than (*k*-1) objects in $N_{k}(x)$
**then** 4:  input *p* into $N_{k}(x)$ ; 5: **else if**
d(x,p)<k−distance(x) && there are (k-1) objects in $N_{k}(x)$
**then** 6:   remove the farthest neighbor in $N_{k}(x)$; 7:   input *p* into $N_{k}(x)$; 8:   recalculate k−distance(x); 9:   recalculate mk−distance(x); 10:   recalculate $N_{mk}(x)$; 11: **else** break; 12: **end if**
 13: **end for**

**Figure 1:**
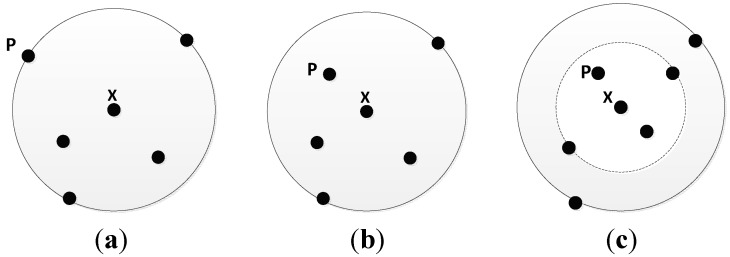
(**a**) Situation 1; (**b**) Situation 2; (**c**) Situation 3.

According to the different positions which *p* inserts into, the influence of *N_k_*(*x*) differs. There are three different cases as follows: 

**Situation 1.** In Figure 1a, *d*(*x*, *p*) = *k-distance*(*x*), that is *p* falls on the circle of the *k*-distance neighborhood of object *x*. Put *p* into *N_k_*(*x*) directly.

**Situation 2.** In Figure 1b, *d*(*x*, *p*) < *k-distance*(*x*), that is *p* falls within the circle of the *k*-distance neighborhood of *x*. If there are less than (*k*-1) objects within the circle, then put *p* into *N_k_*(*x*).

**Situation 3.** In Figure 1c, *d*(*x*, *p*) < *k-distance*(*x*), *p* falls within the circle of the *k*-distance neighborhood of *x*. If there are exactly (*k* − 1) objects within the circle, firstly remove the farthest neighbor in *N_k_*(*x*), then put *p* into *N_k_*(*x*) and recalculate *k-distance*(*x*).

After updating of *N_k_*(*x*) and *k-distance*(*x*), $N_{mk}(x)$ and *mk-distance*(*x*) can also be recalculated.

Algorithm 5 describes the updating process of $lrd_{mk}(x)$ and $lrd_{mk}(y)$, where *x* is a first-level influenced object and *y* is a second-level influenced object. Algorithm 6 describes the updating process of $LOF_{mk}\left(x\right)$, $LOF_{mk}\left(y\right)$, and $LOF_{mk}\left(z\right)$, where *x* is a first-level influenced object, *y* is the second-level influenced object and *z* is the third-level influenced object.

**Algorithm 5.** Update LRD ($F_{1}(p)$, $F_{2}(p)$)0: **for** all objects *x* in $F_{1}(p)$
**do** 1:  recalculate $lrd_{mk}(x)$; 2: **end for** 3: **for** all objects y in $F_{2}(p)$
**do** 4:  recalculate $lrd_{mk}(y)$; 5: **end for**

**Algorithm 6.** Update LOF ($F_{1}(p)$, $F_{2}(p)$, $F_{3}(p)$)0: **for** all objects *x* in $F_{1}(p)$
**do** 1: recalculate $LOF_{mk}\left(x\right)$; 2: **end for** 3: **for** all objects *y* in $F_{2}(p)$
**do** 4: recalculate $LOF_{mk}\left(y\right)$; 5: **end for** 6: **for** all objects *z* in $F_{3}(p)$
**do** 7: recalculate $LOF_{mk}\left(z\right)$; 8: **end for**

### 3.2. The Fuzzy Linear Regression Process

Considering the possible anomalies of sensed readings, and the uncertain relationships among them, it’s quite hard to reflect the fuzzy relationships by the simple linear regression which may cause prediction errors between the regression values and the actual sensed values. It’s better to characterize the output variable and regression coefficients by fuzzy numbers.

For medical sensor readings, we consider that the normal sensor readings from several days ago always show similarities, whereas abnormal sensor readings of adjacent times may deviate from each other, so the physiological parameters of a patient at a given time are closely associated with the historical data of adjacent times (which may be within several hours) instead of readings from several days ago. It also means that the impacts of historical data on the estimated outputs are more important at nearer monitoring times.

Based on the limitation mentioned above, we propose a linear regression model based on trapezoidal fuzzy number to predict a more appropriate fuzzy value for the suspected reading. In this regression model, we regard the minimum sum of regression error as a new objective function, and propose a method to obscure the sensor data using the expected value of trapezoidal fuzzy number. In addition, our proposed regression model has given adequate consideration to the different impacts of historical sensor data. By constructing the minimum sum of regression error and fuzzifying readings, we achieve more precise estimated outputs.

Construct the following fuzzy linear function:

(9) $$\tilde{Y_{j}}=\tilde{A_{0}}+\tilde{A_{1}}x_{j1}+\tilde{A_{2}}x_{j2}+\cdots +\tilde{A_{n}}x_{jn}$$

where *n* is the number of independent variables, *j* is *j*-th data vector on time *T_j_* which is involved in regression modeling. In Equation (9), the prediction value $\tilde{Y_{j}}$ and regression coefficient$\;\tilde{A_{i}}(i=0,1,\cdots ,n)$ are fuzzy values, and $x_{ji}$ is *i*-th measured real number of *j*-th vector. Define $\tilde{{\text{Y}}_{\text{j}}}$ and $\tilde{A_{i}}$ are trapezoidal fuzzy numbers, so $\tilde{Y_{j}}=(y_{aj},y_{bj},y_{lj},y_{rj})$, $\tilde{A_{i}}=(a_{i},b_{i},l_{i},r_{i})$, the membership function of $\tilde{Y_{j}}$ is defined as follows:

(10) $${\text{μ}}_{y}\left(y_{j}\right)=\{\begin{array}{c} \frac{y_{j}-y_{aj}+y_{lj}}{y_{lj}},y_{aj}-y_{lj}<y_{j}<y_{aj}\\ 1,y_{aj}\leq y_{j}\leq y_{bj}\\ \frac{y_{bj}+y_{rj}-y_{j}}{y_{rj}},y_{bj}<y_{j}\leq y_{bj}+y_{rj}\\ 0,otherwise \end{array}$$

where:

(11) $$y_{aj}=\overset{n}{\underset{i=1}{\sum }}a_{i}x_{ji}+a_{0}$$

(12) $$y_{bj}=\overset{n}{\underset{i=1}{\sum }}b_{i}x_{ji}+b_{0}$$

(13) $$y_{lj}=\overset{n}{\underset{i=1}{\sum }}l_{i}\left|x_{ji}\right|+l_{0}$$

(14) $$y_{rj}=\overset{n}{\underset{i=1}{\sum }}r_{i}\left|x_{ji}\right|+r_{0}$$

These historical sensed readings are real numbers, and must be fuzzified for calculating the corresponding fuzzy regression coefficients $\tilde{A_{i}}(i=0,1,\cdots ,n)$. We construct an optimized prediction model of trapezoidal fuzzy numbers so the prediction value is closer to the true value. To resolve the problem of fuzzifying historical values, we introduce a fuzzy number expectation which is a real number with reflecting the values of fuzzy number in average meaning. The expectation of trapezoidal fuzzy number [16] is:

(15) $$\tilde{{\text{E}}_{\text{A}}}=\frac{2\text{a}+2\text{b}-\text{l}+\text{r}}{4}$$

We construct *u* sets of data to calculate the fuzzy coefficients using measured data. As physiological readings are cyclical, the value of *u* is the total number of readings that pick out faulty data in a period. Assume that there are totally *m* groups of measured samples. The following formula represents the *m*-th sample group in one period:

(16) $$\{\begin{array}{c} {\text{Y}}_{1\text{m}},{\text{x}}_{11},{\text{x}}_{12},\cdots ,{\text{x}}_{1\text{n}}\\ {\text{Y}}_{2\text{m}},{\text{x}}_{21},{\text{x}}_{22},\cdots ,{\text{x}}_{2\text{n}}\\ \vdots \\ {\text{Y}}_{\text{um}},{\text{x}}_{\text{u}1},{\text{x}}_{\text{u}2},\cdots ,{\text{x}}_{\text{un}} \end{array}$$

where *u* is the number of samples in one group, *n* is the number of independent variables. For independent variables $x_{ji}(i=1,2,\cdots ,n,\;j=1,2,\cdots ,u)$ in the *m*-th group, we keep the precise real values unchanged and use them to calculate the fuzzy coefficients. For output variables $Y_{jh}(j=1,2,\cdots ,u,\;h=1,2,\cdots ,m)$, regard the historical measured data at the same instant but in different groups as multi-measured results. Let $y_{bj}$ and $y_{aj}$ be the maximum and minimum of $Y_{jh}(h=1,2,\cdots ,m)$ respectively:

(17) $${\text{y}}_{\text{bj}}=\text{max}\{{\text{Y}}_{\text{j}1},{\text{Y}}_{\text{j}2},{\text{Y}}_{\text{j}3},\cdots ,{\text{Y}}_{\text{jm}}\}$$

(18) $${\text{y}}_{\text{aj}}=\text{min}\{{\text{Y}}_{\text{j}1},{\text{Y}}_{\text{j}2},{\text{Y}}_{\text{j}3},\cdots ,{\text{Y}}_{\text{jm}}\}$$

The highest and lowest dependent variables in the samples are viewed as parameters of *a* and *b*, so only parameters of $y_{lj}$ and $y_{rj}$ corresponding to $\tilde{Y_{jm}}$ are changing, and thus, the output variables $\tilde{Y_{jm}}(j=1,2,\cdots ,u)$ have been fuzzified:

(19) $$\{\begin{array}{c} \tilde{{\text{Y}}_{1\text{m}}},{\text{x}}_{11},{\text{x}}_{12},\cdots ,{\text{x}}_{1\text{n}}\\ \tilde{{\text{Y}}_{2\text{m}}},{\text{x}}_{21},{\text{x}}_{22},\cdots ,{\text{x}}_{2\text{n}}\\ \vdots \\ \tilde{{\text{Y}}_{\text{um}}},{\text{x}}_{\text{u}1},{\text{x}}_{\text{u}2},\cdots ,{\text{x}}_{\text{un}} \end{array}$$

The least square method is a mathematical procedure for finding the best-fitting curve to a given set of points by minimizing the sum of the squares of the residuals of the points from the curve, so we borrow the ideas from the least square method to ensure the minimal sum of differences of evaluation precision R, it can be defined as follows:

(20) $$R=\overset{u}{\underset{j=1}{\sum }}{\left(\frac{E_{\tilde{Y_{jm}}}-Y_{jm}}{Y_{jm}}\right)}^{2}$$

where $Y_{jm}$ is a precise prediction value on *m*-th sample group. Then $E_{\tilde{Y_{jm}}}$ is calculated as follows:

(21) $$E_{\tilde{Y_{jm}}}=\frac{2y_{bj}+2y_{aj}-y_{lj}+y_{rj}}{4}$$

Besides, for each tuple of sensor data, the fuzzy linear regression requires the estimated fuzzy number to contain the observed data with more than the degree of fitting ${\text{λ}}_{j}(0\leq {\text{λ}}_{j}\leq 1)$ which is a constant chosen by the decision-maker:

(22) $${\text{μ}}_{y}\left(y_{j}\right)\geq {\text{λ}}_{j}$$

Namely:

(23) $$\{\begin{array}{c} \frac{y_{j}-y_{aj}+y_{lj}}{y_{lj}}\geq {\text{λ}}_{j},y_{aj}-y_{lj}<y_{j}<y_{aj}\\ \frac{y_{bj}+y_{rj}-y_{j}}{y_{rj}}\geq {\text{λ}}_{j},y_{bj}<y_{j}\leq y_{bj}+y_{rj} \end{array}$$

For different readings, ${\text{λ}}_{j}$ is designed as different membership. In general, the closer to the prediction instant, the more important the readings are, and the higher the corresponding membership is. The parameters to be solved are:

(24) $$Min\;R=\overset{u}{\underset{j=1}{\sum }}{\left(\frac{E_{\tilde{Y_{jm}}}-Y_{jm}}{Y_{jm}}\right)}^{2}$$

(25) $$s.t.\;Y_{jm}=\left(y_{aj},y_{bj},y_{lj},y_{rj}\right)$$

(26) $$y_{aj}=min\{Y_{j1},Y_{j2},Y_{j3},\cdots ,Y_{jm}\}$$

(27) $$y_{bj}=max\{Y_{j1},Y_{j2},Y_{j3},\cdots ,Y_{jm}\}$$

(28) $$y_{aj}=\overset{n}{\underset{i=1}{\sum }}a_{i}x_{ji}+a_{0}$$

(29) $$y_{bj}=\overset{n}{\underset{i=1}{\sum }}b_{i}x_{ji}+b_{0}$$

(30) $$y_{lj}=\overset{n}{\underset{i=1}{\sum }}l_{i}\left|x_{ji}\right|+l_{0}$$

(31) $$y_{rj}=\overset{n}{\underset{i=1}{\sum }}r_{i}\left|x_{ji}\right|+r_{0}$$

(32) $$(\;\overset{n}{\underset{i=1}{\sum }}a_{i}x_{ji}+a_{0})-(1-{\text{λ}}_{j})(\overset{n}{\underset{i=1}{\sum }}l_{i}\left|x_{ji}\right|+l_{0})\leq y_{j}$$

(33) $$(\overset{n}{\underset{i=1}{\sum }}b_{i}x_{ji}+b_{0})+(1-{\text{λ}}_{j})(\overset{n}{\underset{i=1}{\sum }}r_{i}\left|x_{ji}\right|+r_{0})\geq y_{j}\;y_{lj}>0,y_{rj}>0,j=1,2,\cdots ,u,0\leq {\text{λ}}_{j}\leq 1$$

Expectation $E_{\tilde{Y_{jm}}}$ can be linearly described by $y_{lj}$ and $y_{rj}$. The object function *R* is a quadratic function and all of restriction conditions are linear. Obviously, this is a nonlinear programming problem with one single objective function and several linear restrictions. In order to find the optimal solution to this problem and reduce the computation cost, we firstly transfer this constrained nonlinear programming problem into unconstrained nonlinear programming problem.

Consider the restrictions in this nonlinear programming model, Equations (28)–(33) can be rewritten as follows:

(34) $$h_{1}=y_{aj}-\overset{n}{\underset{i=1}{\sum }}a_{i}x_{ji}+a_{0}$$

(35) $$h_{2}=y_{bj}-\overset{n}{\underset{i=1}{\sum }}b_{i}x_{ji}+b_{0}$$

(36) $$h_{3}=y_{lj}-\overset{n}{\underset{i=1}{\sum }}l_{i}\left|x_{ji}\right|+l_{0}$$

(37) $$h_{4}=y_{rj}-\overset{n}{\underset{i=1}{\sum }}r_{i}\left|x_{ji}\right|+r_{0}$$

(38) $$h_{i}=0,i=1,2,3,4$$

(39) $$g_{1}=\;y_{j}-((\;\overset{n}{\underset{i=1}{\sum }}a_{i}x_{ji}+a_{0})-(1-{\text{λ}}_{j})(\overset{n}{\underset{i=1}{\sum }}l_{i}\left|x_{ji}\right|+l_{0}))$$

(40) $$g_{2}=(\overset{n}{\underset{i=1}{\sum }}b_{i}x_{ji}+b_{0})+(1-{\text{λ}}_{j})(\overset{n}{\underset{i=1}{\sum }}r_{i}\left|x_{ji}\right|+r_{0})-y_{j}$$

(41) $$g_{i}=0,i=1,2$$

Then we construct the following unconstrained nonlinear programming model which is equivalent to the original constrained nonlinear programming model:

(42) $$\overset{u}{\underset{j=1}{\sum }}{\left(\frac{E_{\tilde{Y_{jm}}}-Y_{jm}}{Y_{jm}}\right)}^{2}+\overset{4}{\underset{i=1}{\sum }}\left|h_{i}\right|+\overset{2}{\underset{i=1}{\sum }}\left|min(0,g_{i})\right|$$

To find the optimal solution for this model, many traditional solutions have been proposed, such as the Lagrange multiplier method or Conjugate Gradient method. Meanwhile, several heuristic algorithms such as Genetic Algorithms (GA) [17,18,19] also play an important role in solving this problem, because it is an efficient method to deal with the nonlinear programming problem with high-complexity and multi-parameters.

We use a genetic algorithm to get the optimal solution to Equation (42), so as to ensure the minimal sum of differences *R* when each membership of prediction variable is not lower than ${\text{λ}}_{j}$. Then we get the fuzzy representation of historical sensed readings and the least square estimation $\tilde{\hat{A_{i}}}=(\hat{a_{i}},\hat{b_{i}},\hat{l_{i}},\hat{r_{i}})$ that is related to the fuzzy regression coefficients $\tilde{A_{i}}(i=0,1,\cdots ,n)$. The normal data will be involved in the prediction after getting the fuzzy coefficients. Finally, we get the fuzzy prediction value of the given sensor.

### 3.3. Fault Judgment Criterion

We define the following fault judgment criterion. As shown in Table 1, the black trapezoid indicates the prediction result of the fuzzy number $\tilde{Y_{p}}$. λ is the similarity membership degree and 0.5<λ<1. (a−l) and (b+r) are the lower bound and upper bound of the fuzzy number, a and b are corresponding values of the membership degree λ. The red line indicates the normal interval of the corresponding physiological parameter. low and high are the lower bound and upper bound of the normal interval. y indicates the actual sensed readings.

As shown in Table 1, whether a sensor is good or faulty depends on different relative position relationships between the prediction result of the fuzzy number and the normal intervals of the corresponding physiological parameter. In addition, it depends on which interval the actual sensed readings lie in. The undecided state indicates that the state of the corresponding sensor cannot be determined in this detection round because physiological indicators of patients may not synchronously change at the same time. It may be caused by a detection delay or a very slow change of the physiological parameter. We continue to detect the undecided faulty nodes in successive detection rounds. If the undecided state of the same sensor remains unchanged after the following several rounds of detection, then it will be viewed as faulty. The undecided state in judgment rule is suitable for various readings’ out-of-sync status.

**Table 1:** The relationships between $\tilde{Y_{p}}$ and y.

The Relationships between $\tilde{Y_{p}}$ and *Y*	Different Intervals Which the Actual Sensed Readings Lie in	
	*Normal Interval*	*Anomalous Interval*
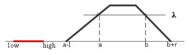	low≤y≤high, fault	y<low, fault *high* < *y* < *a* undecided a≤y≤b good b<y≤b+r, undecided b+r<y, fault
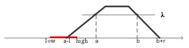	low≤y<a−l, fault a−l≤y≤high, undecided	y<low, fault high<y<a, undecided a≤y≤b, good b<y≤b+r, undecided b+r<y, fault
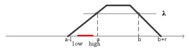	low≤y≤high, undecided	y<a−l, fault a−l≤y<low, undecided high<y<a, undecided a≤y≤b, good b<y≤b+r, undecided b+r<y, fault
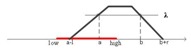	low≤y<a−l, fault a−l≤y<a, undecided a≤y≤high, good	y<low, fault high<y≤b, good b<y≤b+r, undecided b+r<y, fault
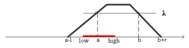	low≤y<a, undecided a≤y≤high, good	y<a−l, fault a−l≤y<low, undecided high<y≤b, good b<y≤b+r, undecided b+r<y, fault
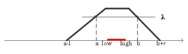	low≤y≤high, good	y<a−l, fault a−l≤y<a, undecided a≤y<low, good high<y≤b, good b<y≤b+r, undecided b+r<y, fault
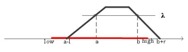	low≤y<a−l, fault a−l≤y<a, undecided a≤y≤b, good b<y≤high, undecided	y<low, fault high<y≤b+r, undecided b+r<y, fault
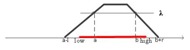	low≤y<a, undecided a≤y≤b, good b<y≤high, undecided	y<a−l, fault a−l≤y<low, undecided high<y≤b+r, undecided b+r<y, fault
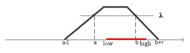	low≤y≤b, good b<y≤high, undecided	y<a−l, fault a−l≤y<a, undecided a≤y<low, good high<y≤b+r, undecided b+r<y, fault
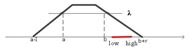	low≤y≤high, undecided	y<a−l, fault a−l≤y<a, undecided a≤y≤b, good b<y<low, undecided high<y≤b+r, undecided b+r<y, fault
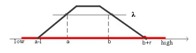	low≤y<a−l, fault a−l≤y<a, undecided a≤y≤b, good b<y≤b+r, undecided b+r<y≤high, fault	y<low, fault high<y
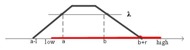	low≤y<a, undecided a≤y≤b, good b<y≤b+r, undecided b+r<y≤high, fault	y<a−l, fault a−l≤y<low, undecided high<y, fault
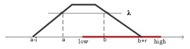	low≤y≤b, good b<y≤b+r, undecided b+r<y≤high, fault	y<a−l, fault a−l≤y<a, undecided a≤y<low, good high<y, fault
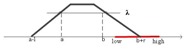	low≤y≤b+r, undecided b+r<y≤high, fault	y<a−l, fault a−l≤y<a, undecided a≤y≤b, good b<y<low, undecided high<y, fault
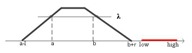	low≤y≤high, fault	y<a−l, fault a−l≤y<a, undecided a≤y≤b, good b<y<low, undecided high<y, fault

We also find that if the actual sensed readings lie in the interval that corresponds to similarity membership degree λ, and then the sensor is regarded as good. If the actual output sensor data is greater than the larger one of (b+r) and high, or less than the smaller one of (a−l) and low, then the sensor is faulty. Otherwise, the sensor is considered as undecided.

## 4. Simulation Results

In this section, we analyze the performance results of DFD-M and typical algorithms. We obtain the variations of physiological readings including Heart Rate (HR), Respiration Rate (RR), Body Temperature (BT) and Oxygen Saturation (SpO2) from medical sensors. The HR fluctuates from 40 to 140 bpm, which may be caused by movements or physiological abnormalities. RR fluctuates around 20 bpm, and is approximately proportional to HR. SpO2 fluctuates from 90 to 100, and only in a rare case that SpO2 is lower than 90. With a few exceptions, BT basically remains stable around 37 °C. To simulate sensor failure, we artificially insert some failure data into the monitoring data.

All the algorithms are implemented in Matlab R2010a. Our simulations are divided into three parts: (1) simulations for the *D*-LOF algorithm compared with the Kernel-LOF (*Ker-*LOF) [20] and the original LOF [15] to verify the detection accuracy rate and time complexity; (2) simulations for fuzzy linear regression based on trapezoidal fuzzy numbers (*FLR-Tra*), simple linear regression (*SLR*) and fuzzy linear regression based on triangle fuzzy numbers (*FLR-Tri*) to demonstrate the evaluation error; (3) simulations for the whole detection performances of *DFD-M,* the fault detection algorithm (*DA-J48*) proposed in [11], and using *FLR-Tri* and *SLR* to replace the regression model in our proposed algorithm.

### 4.1. Simulations for LOF Values

**Figure 2:**
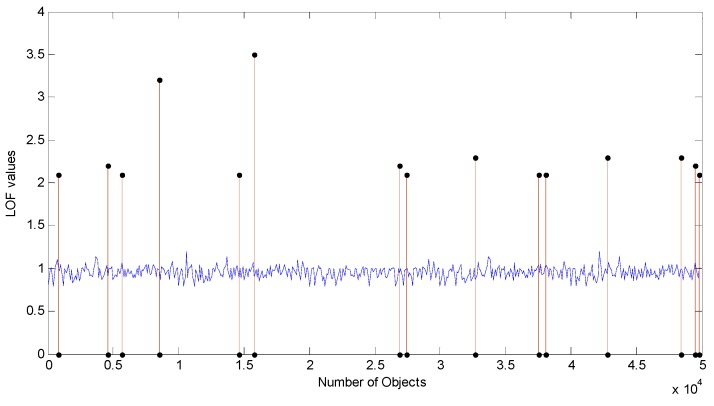
LOF values of objects with 15 outliers (*k* = 10).

In this part, we show the results of detection accuracy rate and time complexity. To simulate the dynamic increment of dataset, 83% instances are in the initial knowledge base, and 17% are viewed as new updated objects. In Figure 2, the simulation settings are *k* = 10 (in the denotation *k*-*distance*), and the maximal size of dataset is 5 ×10^4^ with 15 outliers. As a given threshold of ${\text{LOF}}_{mk}$ is 2.0, LOF values of most of instances fluctuate around 1.0. The 15 instances whose LOF values are greater than 2.0 can be completely detected.

**Figure 3:**
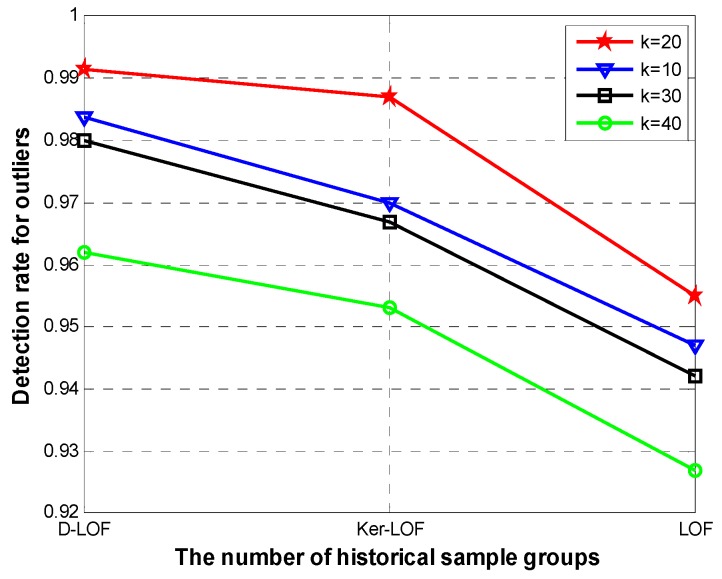
Detection rate for outliers with different *k* values.

As parameter *k* (in the notation *k-distance*) influenced the performance of algorithms, the Figure 3 shows the detection rate for outliers with different *k* when size of dataset is 2 ×10^4^ and the number of outliers is 40. When setting *k* = 20, *D-*LOF has a perfect detection rate with 99.1%, while *Ker-*LOF is 98.7% and original LOF is 95.5%. Changing values of *k*, the detection rates for outliers accordingly decline. But our algorithm still remains a higher performance compared with others.

**Figure 4:**
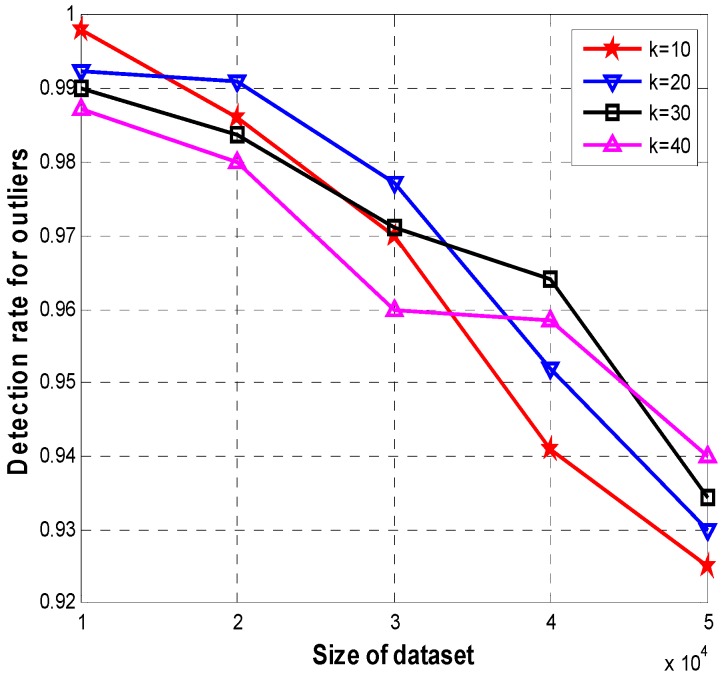
Detection rate for outliers with different *k*.

To further analyze the influences caused by *k* value and the sizes of datasets, we set datasets be two-dimensional instances whose sizes are respectively 0.5, 1, 1.5, 2, 2.5, 3, 3.5, 4, 4.5, 5 with ×10^4^, while there are 25, 35, 45, 55, 65, 75, 85, 95, 105, 115 outliers randomly distributed in these datasets respectively. Figure 4 shows the detection rates for outliers with different *k* when increasing the sizes of datasets in our algorithms. The optimized relations with the highest detection rates are respectively: 1 ×10^4^ with *k* = 10, 2 ×10^4^ with *k* = 20, 2 ×10^4^ with *k* = 20, 4×10^4^ with *k* = 30, and 5×10^4^ with *k* = 40. And the detection rates are respectively 99.8%, 99.1%, 97.7%, 96.3% and 94.2% in these situations. For example, the best rates are respectively 2.3% and 1.8% higher than the worst rates in situations of 4×10^4^ with *k* = 30 and 5×10^4^ with *k* = 40.

**Figure 5:**
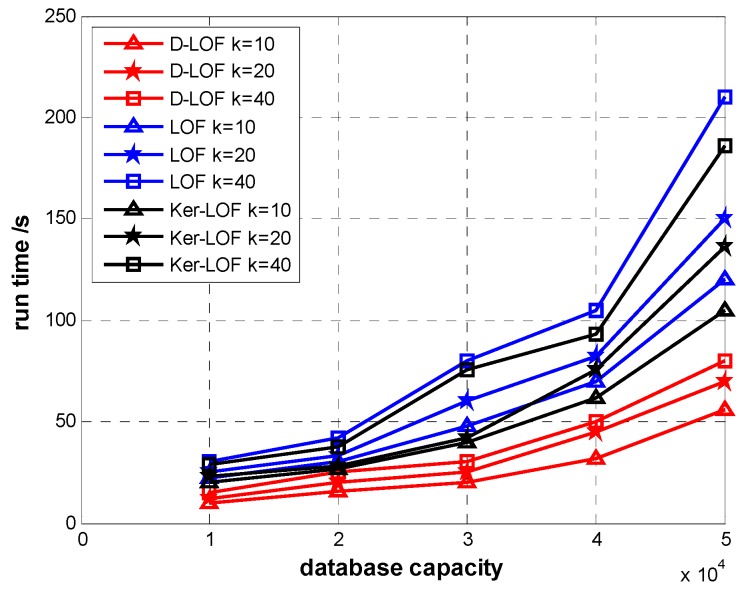
Run time with different *k.*

Figure 5 shows the running time of *D-*LOF, *Ker-*LOF and original LOF. We vary the values of parameter *k* related to the number of neighbors in the domain of an object. The time taken by these algorithms increases as the size of dataset increases, while *D-*LOF has a much lower running time than others. When *k* = 10 and the size of dataset is 5 ×10^4^ with 105 outliers, run time of *D-*LOF is 56 s, which is 53.3% lower than that of original LOF, and 49.2% lower than that of *Ker-*LOF. With the same dataset size, when *k* = 40, running time of *D-*LOF is 81 s. It is 61.9% lower than that of original LOF, and 56.7% lower than *Ker-*LOF. The time complexity is sharply reduced because only three levels of influenced objects update their LOF values. We also find that when the size of dataset is unchanged, the smaller the value of *k* is, the faster the outliers are detected out.

**Figure 6:**
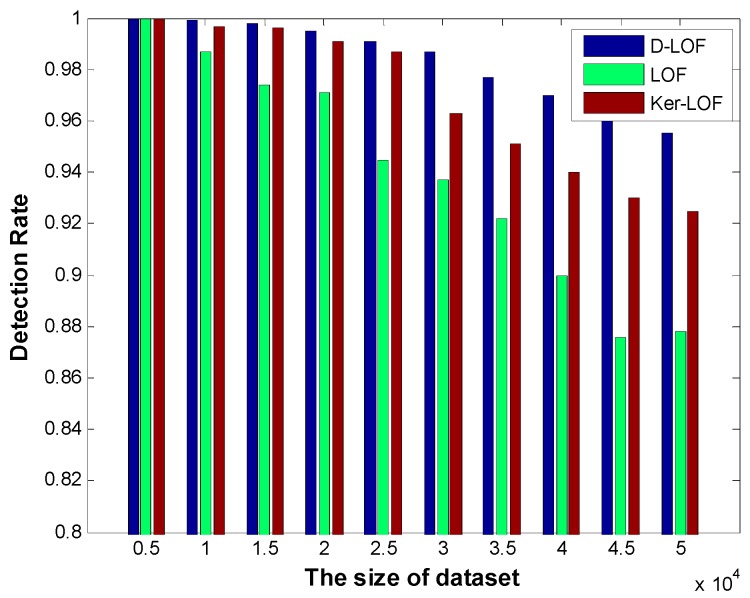
Detection rate with different optimized *k.*

Figure 6 shows the detection rate of *D-*LOF compared with *Ker-*LOF and original LOF when the values of *k* are set as the best optimized according to Figure 3 and Figure 4. When the size of the dataset is smaller, they almost achieve 100% detection rate. As the size of dataset is increasing, all of detection rates slowly decline. Because *D-*LOF is more sensitive to outliers, it is clear that of *D-*LOF performs better than the other two and achieves a higher detection rate for different dataset sizes. Even though the size of the dataset is 5 ×10^4^ with 115 outliers, 109 outliers can be detected by *D-*LOF with a detection rate 94.8%, while that of the original LOF is 87.8%, and for *Ker-*LOF it is 92.4%.

### 4.2. Simulations for Evaluation Error R

**Figure 7:**
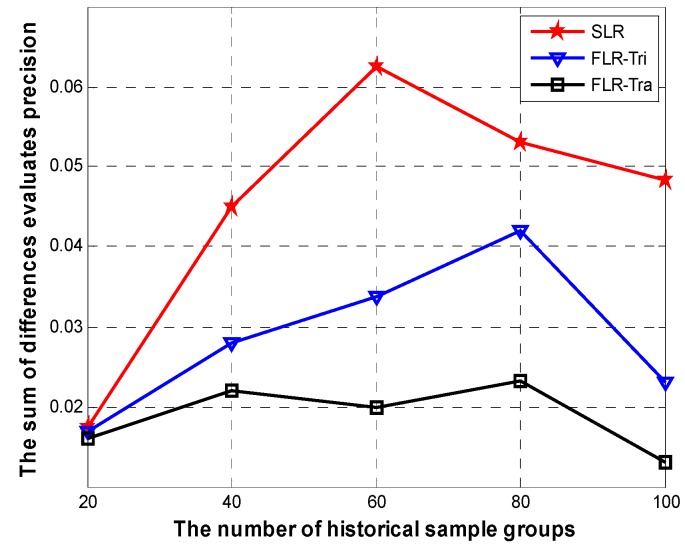
The sum of differences of evaluation precision R.

In this part, we calculate the differences of evaluation precision for *FLR-Tra* in our algorithm compared with *SLR* and *FLR-Tri.* We represent the sum of differences evaluates precision R (defined in III. B section) of the three regression models in Figure 7. It shows the trends from rising to decline with the increasing number of historical sample groups in the linear regression model. That is because, in the initial stage, an increase in the number of historical samples leads to a significant increase of R. When the number of historical sample is 20, the three regression models achieve similar evaluation precision differences, that is 0.0175, 0.0168, and 0.0162 of *SLR*, *FLR-Tri* and *FLR-Tra.* The *FLR-Tra* achieves the top point when the number of samples is 80. When the number of historical samples is 100, the sum of differences precision evaluation of *FLR-Tra* reduces to 0.0143, which is 39.1% lower than that of *FLR-Tri* and 70.8% lower than that of *SLR*. That is, as the number of historical samples becomes even larger, the fuzzified historical values get closer to their actual values, make our regression more accurate, and then R declines.

**Figure 8:**
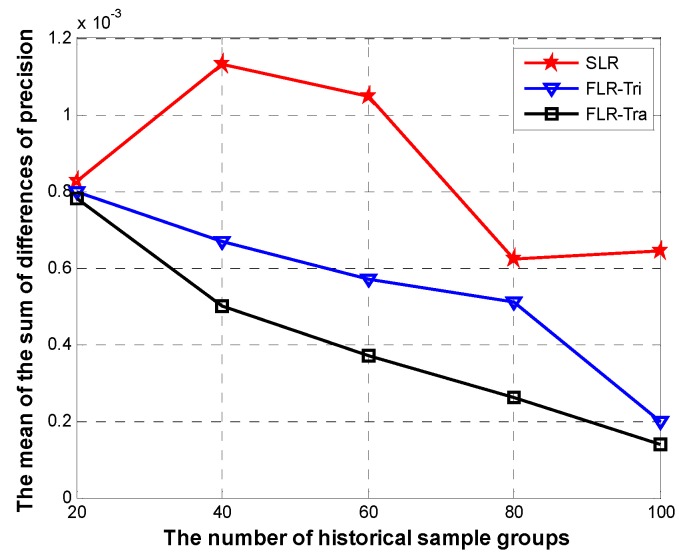
The mean of the sum of difference of evaluation precision R.

In Figure 8, the mean of the sum of differences evaluates precision $\bar{R}$ has an obvious decrease for *FLR-Tra* and *FLR-Tri*, while that of *SLR* rises at the initial stage, and then declines sharply. *FLR-Tra* achieves the best performance on $\bar{R}$.

### 4.3. Simulations for Whole Detection Performances of DFD-M

**Figure 9:**
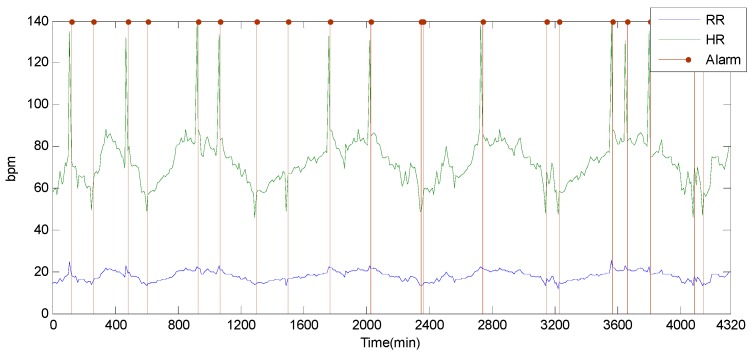
Undetected alarms.

**Figure 10:**
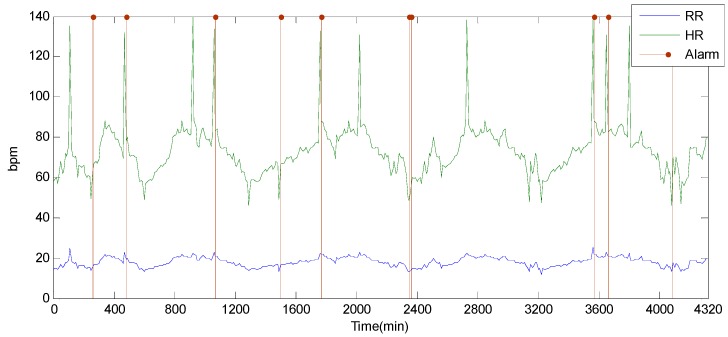
Alarms by DFD-M.

Alarms are reported when readings exceed the normal range. The main task of *DFD-M* is to distinguish data failure from alarms. To simplify for visualization, we only adopt readings of RR and HR in the results of Figure 9, which shows the alarm reporting scene without fault detection. There are in total 19 alarms raised presented in vertical red lines. It reports exceptions for HR and RR. Figure 10 shows the alarms reported by *DFD-M*. Only nine alarms remain in this chart, which excludes the other 10 alarms caused by sensor data faults.

**Figure 11:**
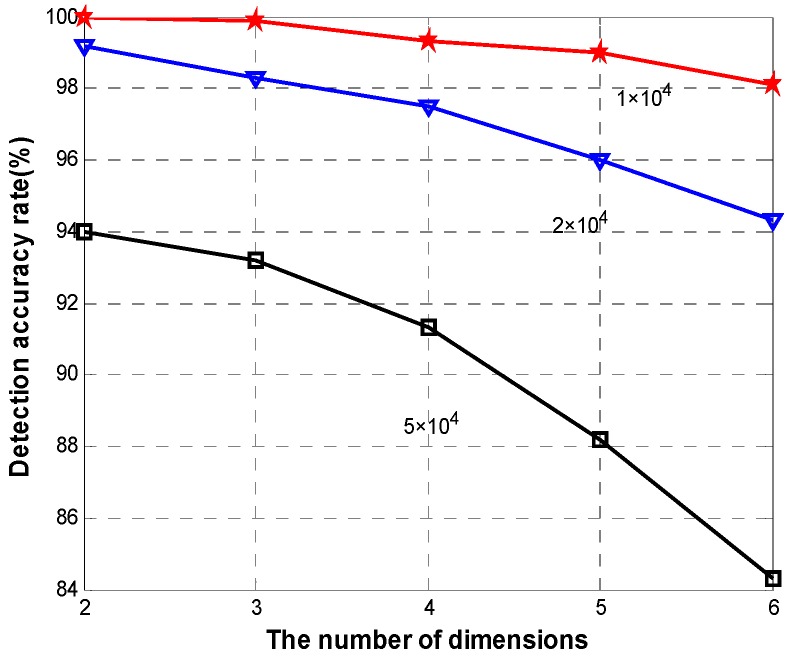
Detection accuracy rate for different data sizes of DFD-M.

**Figure 12:**
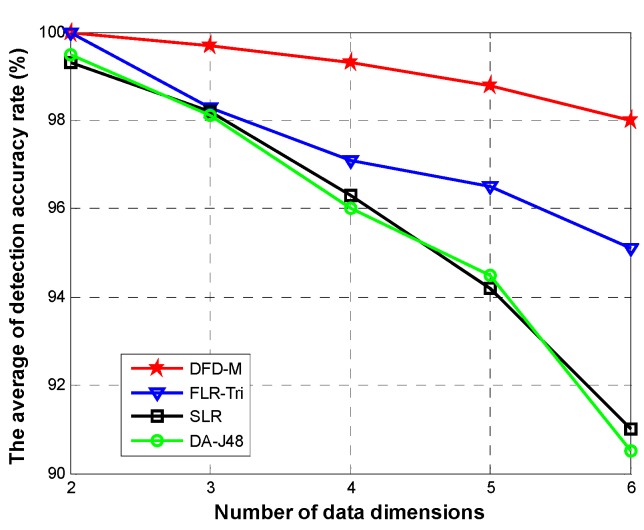
Detection accuracy rate.

To further evaluate the performance of *DFD-M*, we add new two types of readings: blood glucose and blood pressure. When the size of the data is increasing, false alarms inevitably happen. In this part, we mainly analyze the detection accuracy rate and false alarm rate. The latter is the ratio of number of real readings that are misjudged as false to the sum of readings. Figure 11 shows the detection accuracy rate for different data sizes when varying the data dimensions. The smaller the dataset size and dimension are, the higher the detection rate is. The lowest accuracy is 84.6% with 6 dimensions and data size of 5 ×10^4^. Figure 12 shows the average of detection accuracy rate with increasing dimensions. *DFD-M* achieves almost 100% with two data attributes. With the increasing data dimensions, all of the accuracy rates of these algorithms decline slightly. When adding up to six dimensions, the average accuracy rate of *DFD-M* reaches a high level with 97.2%. We use *FLR-Tri* and *SLR* to replace the regression model in our proposed algorithm. The detection accuracy using *FLR-Tri* is 95.1% and only 91.3% in *SLR* with six dimensions. While *DA-J48* has the lowest detection rate 90.5%.

**Figure 13:**
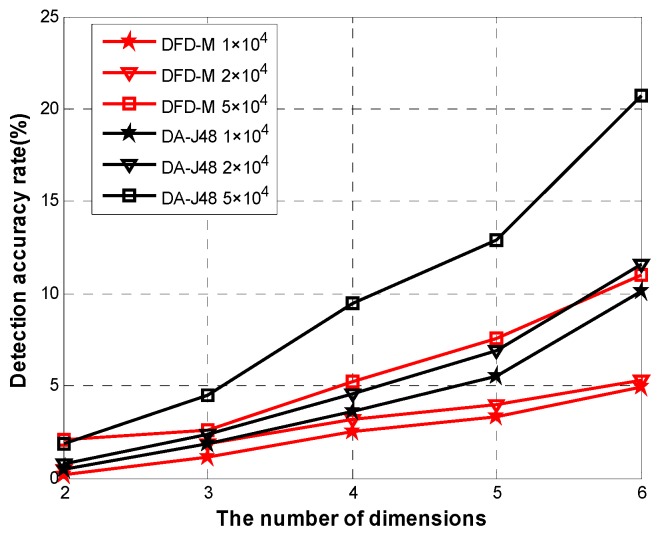
False alarm rate for different data sizes.

Figure 13 shows the false alarm rate for different data sizes when varying data dimensions. The averages of false alarm rates of *DFD-M* are respectively 2.33%, 3.06%, and 5.58% for different data sizes, while that of *DAJ-48* are respectively 4.3%, 6.26% and 9.8%. 

**Figure 14:**
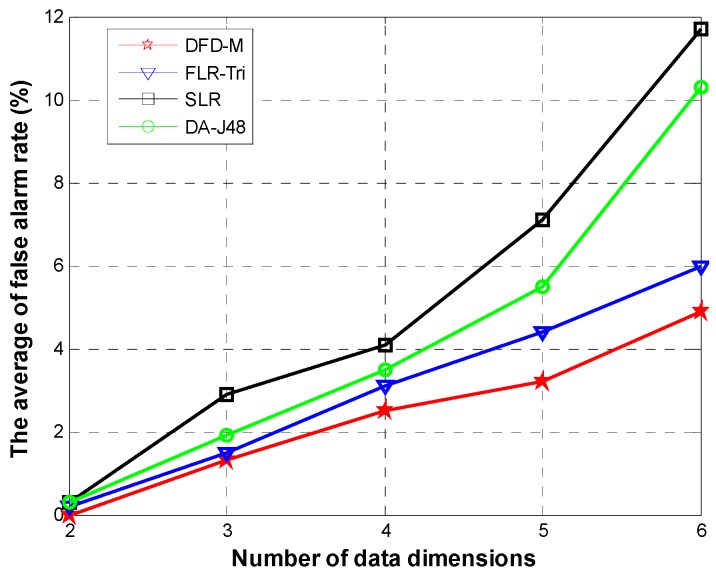
False alarm rate.

Even though the dimensions increase to six and the size of dataset is 5 ×10^4^, *DFD-M* still has a lower false alarm rate of 11.2%, 45.3% lower than that of *DAJ-48*. Figure 14 shows the average of false alarm rates for the abovementioned four algorithms. When adding dimensions, the false alarm rates of these four algorithms increase slowly. When adding up to five dimensions, the false alarm rate of *DFD-M* is only 3.2%, while that of *FLR-Tri* is 4.4%, *SLR* is 7.1% and *DA-J48* is 5.5%. When adding up to six dimensions, the false alarm rate of *DFD-M* is 4.9%, which is 18.3% lower than that of *FLR-Tri*, 55.5% lower than that of *SLR*, and 50.4% lower than that of *DA-J48*. The above experimental results show that *DFD-M* achieves more superior performance than the others.

## 5. Conclusions

The paper proposes a medical sensor fault detection mechanism for data failure called DFD-M. It firstly identifies outlying data vectors, and then uses an improved fuzzy linear regression model to predict the reasonable range for outlying data, and finally it analyzes the relationships between the fuzzy prediction results and the normal intervals by using a novel fault state judgment criterion. The simulations demonstrate that DFD-M has a higher detection accuracy rate and lower false alarm rate that other similar algorithms.
